# Potential impact of atmospheric heating over East Europe on the zonal shift in the South Asian high: the role of the Silk Road teleconnection

**DOI:** 10.1038/s41598-020-63364-2

**Published:** 2020-04-16

**Authors:** Sixian Cen, Wen Chen, Shangfeng Chen, Yuyun Liu, Tianjiao Ma

**Affiliations:** 1 Center for Monsoon System Research, Institute of Atmospheric Physics, Chinese Academy of Sciences, Beijing, China; 2 College of Earth and Planetary Sciences, University of the Chinese Academy of Sciences, Beijing, China; 3 Chengdu Institute of Plateau Meteorology, China Meteorology Administration, Chengdu, China; 4 Heavy Rain and Drought-Flood Disasters in Plateau and Basin Key Laboratory of Sichuan Province, Chengdu, China

**Keywords:** Climate sciences, Mathematics and computing

## Abstract

It is well recognized that the zonal shift in the South Asian High (SAH) has pronounced influences on weather and climate anomalies over surrounding and teleconnected regions. Hence, it is of great importance to investigate the factors related to the interannual variation in the zonal location of the SAH. This study indicates that the anomalous atmospheric apparent heat source (*<Q*_1_>) around East Europe has a close relationship with the interannual variation in the SAH zonal shift during boreal summer. In particular, when above (below) normal *<Q*_1_> exists, the SAH tends to shift westward (eastward). Above-normal *<Q*_1_> over East Europe can trigger an eastward propagating wave train along the subtropical jet stream, resembling the negative phase of the Silk Road teleconnection pattern, with positive geopotential height anomalies around the Iranian Plateau and Northeast Asia and negative anomalies around East Europe and the Tibetan Plateau, which could lead to a westward shift in the SAH. Our model experiments confirm that anomalous *<Q*_1_> around East Europe can exert pronounced impacts on the zonal shift in the SAH by inducing an eastward propagating atmospheric wave train.

## Introduction

The South Asian High (SAH) is the strongest and steadiest anticyclonic circulation system, apart from the polar vortex, in the upper troposphere in boreal summer, and it is one of the members of the Asian summer monsoon system^1,2^. The establishment of the SAH and the associated meridional movement of the ridgeline have close relationships with the onset of the Asian summer monsoon^3–5^ and location of rain belt over East China^6,7^. Changes in the SAH have notable impacts on the weather and climate in many regions, such as precipitation over China^8–11^, India^12–14^, and the Korean peninsula^15–18^. The SAH can also impact precipitation and surface temperature over the North Pacific and North American regions by modulating atmospheric teleconnection patterns^19^. Hence, it is crucial to improve our understanding of SAH variability and its underlying driving factors^20–25^, which may have important implications for regional climate predictions.

The SAH has pronounced variations in terms of location and intensity. The variation in SAH intensity is influenced by Tibetan Plateau heating^26–28^, the Asian summer monsoon^13,29^, the Indian ocean sea surface temperature (SST)^20,21,30^ and SSTs in the tropical central-eastern Pacific^23,24^. For example, the summer SAH tends to be stronger than normal when an El Niño occurs in the preceding winter. This is because a winter El Niño leads to SST warming over the tropical Indian Ocean during the following summer, which further results in a stronger than normal SAH via a Matsuno-Gill-like atmospheric response^20,23,30^.

Changes in the location of the SAH include meridional and zonal shifts. For the meridional shift in the SAH, studies have indicated that the center of the SAH would shift southward (northward) when SST warming (cooling) appears in the Indian Ocean^20,23^. For the zonal shift, Yang *et al*.^31^ suggested that the variation in the SAH zonal location is significantly impacted by SST anomalies in the tropical Indian Ocean and Pacific at the interannual timescale. In particular, the SAH would shift southeastward when SST warming appears in the tropical western Indian Ocean and cooling occurs in the tropical eastern Indian Ocean. In addition, Wei *et al*.^29^ indicated that enhanced condensation heating over the northern Indian peninsula resulting from a stronger Indian summer monsoon could lead to a westward shift in the SAH, with the center being located near the Iranian plateau. At the subseasonal time scale, Yang *et al*.^32^ indicated that the zonal shift in the SAH is closely related to the southward movement of intraseasonal perturbations originating from midlatitudes. In addition, Ren *et al*.^33^ suggested that eastward extension of the SAH on the sub-seasonal timescale is closely related to an eastward propagation of atmospheric wave train over Eurasia. Ren *et al*.^33^ further defined an index as the sub-seasonal anomalies of 200 hPa geopotential height over subtropical eastern Asian to describe eastward extension of the SAH.

As a warm high-pressure system, the establishment, maintenance and variations in the SAH are closely related to the anomalous sensible heating around the Tibetan Plateau^34–37^ and anomalous latent heating associated with the Asian summer monsoon^26,38,39^. For instance, Qian *et al*.^39^ pointed out that the center of the SAH has the heat preference property, which is usually located over or moving toward an area with larger heating rates. Wang *et al*.^40^ found that the SAH tends to shift westward during the peak phase of the 10–20 day low-frequency oscillation of the eastern plateau heat source. Zhang *et al*.^9^ suggested that condensational heating released by east Asian summer monsoon precipitation over the eastern Tibetan Plateau-Yangtze River valley plays an important role in the interannual variation in SAH intensity. These studies all suggested that diabatic heating is important for SAH variations.

The above studies suggested that zonal variations in the SAH were impacted by factors from both the tropics and mid-high latitudes. However, previous studies regarding impact of mid-high latitude systems on the zonal shift of the SAH mainly focused on the subseasonal time scale. In this study, we concentrate on investigating impact of the climate systems over midlatitudes on the zonal shift of the SAH on the interannual time scale. Identifying the contributions of the climate systems over mid-high latitudes to interannual variations in the SAH zonal shift has important implications for regional climate prediction. In this study, we will show that diabatic heating around East Europe has a close relationship with the zonal shift in the SAH at the interannual timescale based on both observational analysis and model experiments. This study will also investigate the physical process behind the impact of diabatic heating around East Europe on the zonal shift in the SAH.

The structure of this study is organized as follows. Section 2 introduces the datasets, methods and model experiments used in this study. Section 3 presents the main results of this study. In this section, we will first provide observational evidence to reveal the close relationship between the zonal shift in the SAH and atmospheric apparent heat source (<*Q*_1_>) around East Europe. Then, we analyze the underlying mechanism linking diabatic heating anomalies around East Europe with the zonal shift in the SAH. Finally, we perform a linear baroclinic model experiment to verify the process behind the impact of the East European diabatic heating anomalies on the zonal shift in the SAH. The primary findings and discussion of this study are presented in section 4.

## Data, methods and model

### Data

The monthly mean air temperature, horizontal winds, vertical velocity, surface pressure, and geopotential height are derived from the National Centers of Environmental Prediction/National Centers of Atmospheric Research (NCEP/NCAR) reanalysis data, with a horizontal resolution of 2.5° × 2.5° and spanning from 1948 to 2019. This study focuses on boreal summer, which refers to the three months of June, July, and August. In addition, this study focuses on interannual variations; thus, following previous studies^41,42^, all variables are subjected to a nine-year Lanczos high-pass filter^43^.

### Methods

The apparent heat source *Q*_1_^44^ is calculated as follows:

1 $${{Q}}_{1}={c}_{p}\left[\frac{\partial T}{\partial t}+\overrightarrow{V}\bullet \nabla T+{\left(\frac{p}{{p}_{0}}\right)}^{k}\omega \frac{\partial \theta }{\partial p}\right]$$

where *T*, $\theta$, $\omega$, and $\overrightarrow{V}$ represent the air temperature, potential temperature, vertical *p*-velocity, and horizontal wind, respectively. *k* = *R*/*c*_*p*_*, R* and *C*_*p*_ are the gas constant and the specific heat at the constant pressure of dry air, respectively, and *p*_0_ is equal to 1000 hPa. The heat source of the whole air column is calculated through the vertical integration of *Q*_1_ from *P*_*t*_ to *P*_*s*_, and *P*_*s*_ and *P*_*t*_ are the surface pressure and 100 hPa pressure, respectively. The <*Q*_1_> is calculated as follows:

2 $$\langle {Q}_{1}\rangle =\frac{1}{g}{\int }_{{p}_{t}}^{{p}_{s}}{{Q}}_{1}dp=\frac{{c}_{p}}{g}{\int }_{{p}_{t}}^{{p}_{s}}\left[\frac{\partial T}{\partial t}+\overrightarrow{V}\bullet \nabla T+{\left(\frac{p}{{p}_{0}}\right)}^{k}\omega \frac{\partial \theta }{\partial p}\right]dp$$

This study employs the wave activity flux proposed by Takaya and Nakamura^45^ to describe the propagation of atmospheric Rossby waves. The wave activity flux can be written as follows:

3 $$W=\frac{1}{2|\overline{U}|}[\begin{array}{c}\overline{u}({\psi {\prime} }_{x}^{2}-\psi {\prime} {\psi {\prime} }_{xx})+\overline{v}({\psi {\prime} }_{x}{\psi {\prime} }_{y}-\psi {\prime} {\psi {\prime} }_{xy})\\ \overline{u}({\psi {\prime} }_{x}{\psi {\prime} }_{y}-\psi {\prime} {\psi {\prime} }_{xy})+\overline{v}({\psi {\prime} }_{y}^{2}-\psi {\prime} {\psi {\prime} }_{yy})\end{array}]$$

where $\psi$ represents streamfunction, $u$ is the zonal wind, and $v$ is the meridional wind. The overbars represent the climatological mean, and primes represent perturbations.

### Model

The linear baroclinic model (LBM) used in this study was developed by the Atmosphere and Ocean Research Institute (AORI), University of Tokyo, and the National Institute for Environmental Studies (NIES)^46,47^. The horizontal resolution of this model is spectral triangular truncation at wavenumber 42 (T42), with 20 vertical levels on a sigma coordinate system. The model includes the horizontal and vertical diffusion. The horizontal diffusion employs biharmonic diffusion, with a time scale of 6 h for the smallest-scale wave. The Rayleigh friction and Newtonian damping is applied in the model, and it have a timescale of (0.5 day)^−1^ for *σ* ≥ 0.9 and (1 day)^−1^ for *σ* ≤ 0.03, while (20 day)^−1^ between them. The initial perturbation set in the line will be ignored. The basic state and external forcing of the LBM experiment are obtained from the NCEP/NCAR reanalysis, and a dry model and time integration method is employed. The length of the time integrated was set to 30 days, and the quasi-steady state was approached after day 15.

## Main results

### Interannual variation in the SAH zonal shift

**Figure 1:**
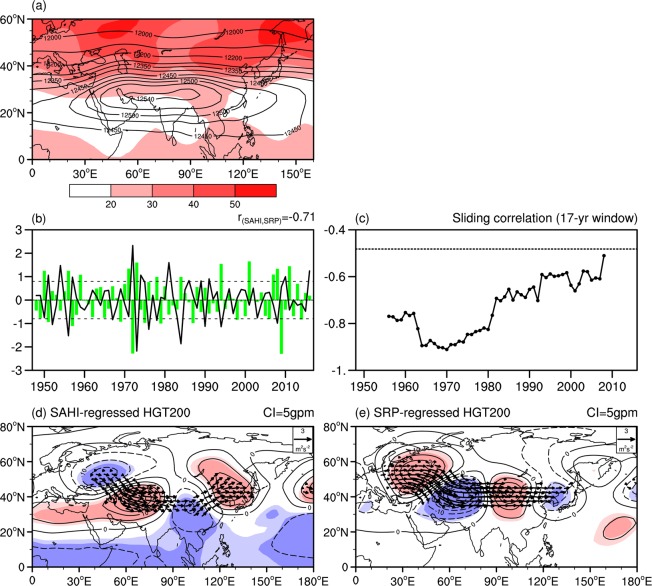
(**a**) Climatology (contour, unit: gpm) and standard deviation (shaded, unit: gpm) of summer 200 hPa geopotential height during 1948–2016. (**b**) Normalized time series of the South Asian High (SAH) index (green bar) and the Silk Road teleconnection pattern (SRP) index (black line). (**c**) Sliding correlation between the SAH index and the SRP index at the central year of a 17-yr window. (**d**) Regression of the 200 hPa geopotential height anomalies (black contour, where the contour interval (CI) is 5 gpm) and the wave activity flux (vectors, unit: m^2^ s^−2^) on the summer SAH index. (**e**) Regression of the 200 hPa geopotential height anomalies (black contour, where the CI is 5 gpm) and the wave activity flux on the SRP index. The horizontal dashed line indicates the 95% confidence level of correlation in (**c**). The dark (light) shadings in (**d,e**) indicate geopotential height anomalies significant at the 99% (95%) confidence level. Wave activity fluxes are omitted when both directions are less than 0.5 m^2^ s^−2^ in (**d,e**). This Figure is created by the NCL^58^ v6.4.0 (http://www.ncl.ucar.edu/).

The climatology and standard deviation of the summer 200 hPa geopotential height for the period of 1948–2016 are shown in Fig. 1a. The oval-shaped SAH is clearly observed over the middle-lower latitudes of Eurasian continent and northeast part of Africa. The 12500 gpm contour extends from 20°N to 35°N and from 30°E to 120°E. Center of the SAH is located over southern slope of the Iranian Plateau and Tibetan Plateau. Meanwhile, large standard deviations with values exceeding 20 gpm are mainly located to the north of 25°N. There are two centers of large standard deviations with value above 50 gpm over East Europe and Okhotsk Sea, respectively.

To describe the zonal shift in the SAH, an east-west shift index of the SAH (SAHI) is first defined as the difference in the standardized geopotential height anomalies at 200 hPa during summer between the regions 22.5°–32.5°N, 55°–75°E and 22.5°–32.5°N, 85°–105°E following the method proposed by Wei *et al*.^29^. The positive (negative) phase of the SAHI corresponds to a westward (eastward) shift in the SAH. Figure 1b displays the normalized time series of the summer SAHI (green bar). Figure 1d shows the regression of the summer 200 hPa geopotential height on the SAHI. A clear atmospheric wave train can be observed over the midlatitudes of Eurasia, with positive geopotential height anomalies over the Iranian Plateau and northeast Asia and negative anomalies around East Europe, Southwest China and South Asia (Fig. 1d). This atmospheric wave train originated from East Europe, which can be confirmed by the wave activity flux (Fig. 1d). This suggests that atmospheric conditions over East Europe may be an important source for the interannual variation in the SAH zonal shift. In addition, the atmospheric wave train associated with the SAHI bears a close resemblance to that related to the Silk Road teleconnection pattern (SRP)^48–50^. To confirm this, following previous studies^48–50^, we define a SRP index as the principal component time series corresponding to the first empirical orthogonal function (EOF) of the summer 200 hPa meridional wind over 20°–60°N, 30°–130°E (Fig. 1b, black line). Figure 1e displays the regression of the summer 200 hPa geopotential height and wave activity flux on the summer SRP index. An obvious atmospheric wave train can be found over the subtropics of Eurasia, propagating along the subtropical jet stream (Fig. 1e), which is similar to that related to the negative phase of the SAHI (Fig. 1d), with the pattern correlation between them as high as −0.81. In addition, the temporal correlation coefficient between the summer SAHI and SRP index (SRPI) during 1948–2016 reached −0.71, which was significant at the 99% confidence level. This suggests that the interannual variation in the SRP can explain approximately 49% of the interannual variation in the SAH zonal shift. In particular, most of the extreme SAHI corresponds well with a large value of the SRP index (e.g., in 1950, 1951, 1956, 1957, 1972, 1973, 1978, 1984, 1994, and 2008). This suggests that the pronounced connection of the SAHI with the SRP was not due to a few extreme years. Figure 1c displays 17-year moving correlation coefficients between the SAHI and the SRP index. From Fig. 1c, the SRP index has a significant negative correlation with the SAHI for the entire period, but the correlation shows a decreasing trend after the early-1970s. Results are highly similar when employing other moving windows (e.g., 15, 19, 31, and 33-yr) of running correlations (not shown). Hence, the above evidence suggests that the interannual variation in the summer SRP has a close relationship with the interannual variation in the summer SAH zonal shift. In particular, from Fig. 1e, significant positive geopotential height anomalies appear around west China and significant negative geopotential height anomalies are apparent over central Asia during positive phase of the SRP. Comparison of Fig. 1a,e suggests that during positive (negative) phase of the SRP, center of the summer SAH tends to shift eastward (westward).

Previous studies indicate that the SAH is a warm high. The interannual variation in the SAH zonal location is strongly influenced by diabatic heating^26,27,51,52^. In the following section, the diabatic heating anomalies related to the zonal shift in the SAH are further examined. The positive (negative) phase of the summer SAHI is defined as those years when the normalized SAHI is larger (less) than 0.8 (−0.8). Based on this criterion, thirteen years (i.e., 1950, 1956, 1959, 1971, 1973, 1976, 1978, 1984, 1994, 2001, 2007, 2008, and 2011) are selected as defining the positive phase of the SAHI. In addition, twelve years (1949, 1951, 1957, 1969, 1972, 1975, 1979, 1987, 1998, 2006, 2009, and 2014) are identified as the negative phase of the SAHI.

**Figure 2:**
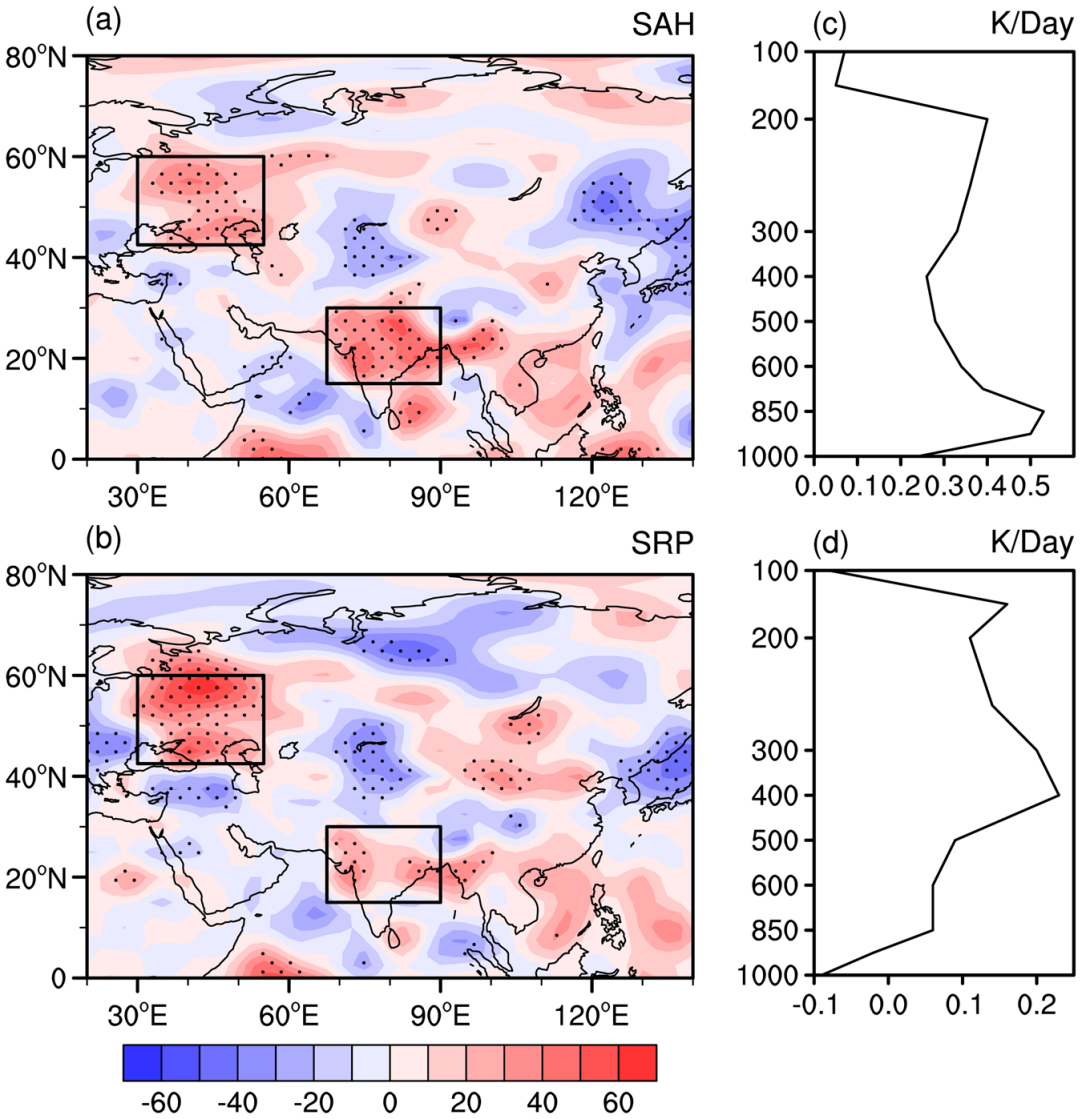
(**a**) Composite anomalies of the <*Q*_1_> (W m^−2^) between the positive and negative phases of the SAH index. (**b**) Composite anomalies of the <*Q*_1_> (W m^−2^) between the negative and positive phases of the SRP index. (**c**) Composite anomalies of vertical profile of the <*Q*_1_> averaged over the East Europe between the positive and negative phases of the SAH index. (**d)** Composite anomalies of vertical profile of the <*Q*_1_> averaged over the Indian subcontinent between the positive and negative phases of the SAH index. The definition of <*Q*_1_> was provided in the text. Anomalies that are significantly different from zero at the 95% confidence level in (**a,b**) are marked with black dots. The two black boxes over East Europe (42.5°−60°N, 30°–55°E) and Indian subcontinent (15°–30°N, 67.5°–90°E) in (**a,b**) were employed to construct the region-averaged <*Q*_1_> index in Fig. 3. This Figure is created by the NCL^58^ v6.4.0 (http://www.ncl.ucar.edu/).

Figure 2a shows the composite difference of <*Q*_1_> between the positive and negative phases of the SAHI. Pronounced positive <*Q*_1_> anomalies are apparent around the northern Indian Peninsula, with an amplitude of approximately 60 W m^−2^. Previous studies have demonstrated that atmospheric heating anomalies around India can exert significant impacts on the variation in the Silk Road teleconnection pattern^53^. In addition, Wei *et al*.^29^ pointed out that the anomalous <*Q*_1_> over the northern Indian Peninsula has a close relationship with the zonal shift in the SAH. It is noted that marked positive <*Q*_1_> values can also be observed around East Europe, corresponding well to the starting position of the atmospheric wave train associated with the summer SAHI and SRP (Figs. 1d,e and 2a). Yasui *et al*.^54^ demonstrated that anomalous heating in the eastern Mediterranean region is an important source of the SRP. Positive and negative phases of the SRPI are selected according to the 0.8 standard deviation. Based on this criterion, twelve years including 1951, 1954, 1957, 1972, 1974, 1975, 1981, 1986, 1989, 1991, 2010, and 2016 are defined as positive phases of the SRP. In addition, 1950, 1952, 1956, 1967, 1973, 1976, 1978, 1984, 1990, 1994, 2003, and 2008 (also a total of 12 years) are identified as the negative phases of the SRP. Figure 2b shows the composite anomalies of the <*Q*_1_> between the negative and positive phases of the SRP. It shows that spatial distributions of the composited <*Q*_1_> are similar based on the SRP and SAH indices (Fig. 2a,b). This further confirms that the SRP has a close relation with the zonal shift of the SAH. The above evidence implies that the anomalous <*Q*_1_> around East Europe may also play an important role in modulating the interannual variation in the SAH zonal shift, which will be further verified in the following section.

To further analyze the relation of the <*Q*_1_> over East Europe and the northern Indian Peninsula with the zonal shift in the SAH, two <*Q*_1_> indices were defined as the regional mean <*Q*_1_> over the northern Indian Peninsula (IPQ for short) and East Europe (EUQ for short). These two regions were selected according to the results shown in Fig. 2a. Note that the results obtained are very similar for the reasonable change in the regions used to define the IPQ and EUQ. The correlation coefficients of the SAHI with the IPQ and EUQ are 0.54 and 0.51, respectively, and are both significant at the 99% confidence level. Figure 2c,d show composite anomalies of vertical profile of the <*Q*_1_> averaged over East Europe and the northern Indian Peninsula, respectively, between two phases of the SAH index. For East Europe, maximum <*Q*_1_> anomaly appears at the lower troposphere (i.e., 850 hPa) with value of approximately 0.53 K Day^−1^ (Fig. 2c). For the <*Q*_1_> over the northern Indian Peninsula, maximum positive <*Q*_1_> anomaly is observed at 400 hPa with value about 0.23 K Day^−1^ (Fig. 2d).

**Figure 3:**
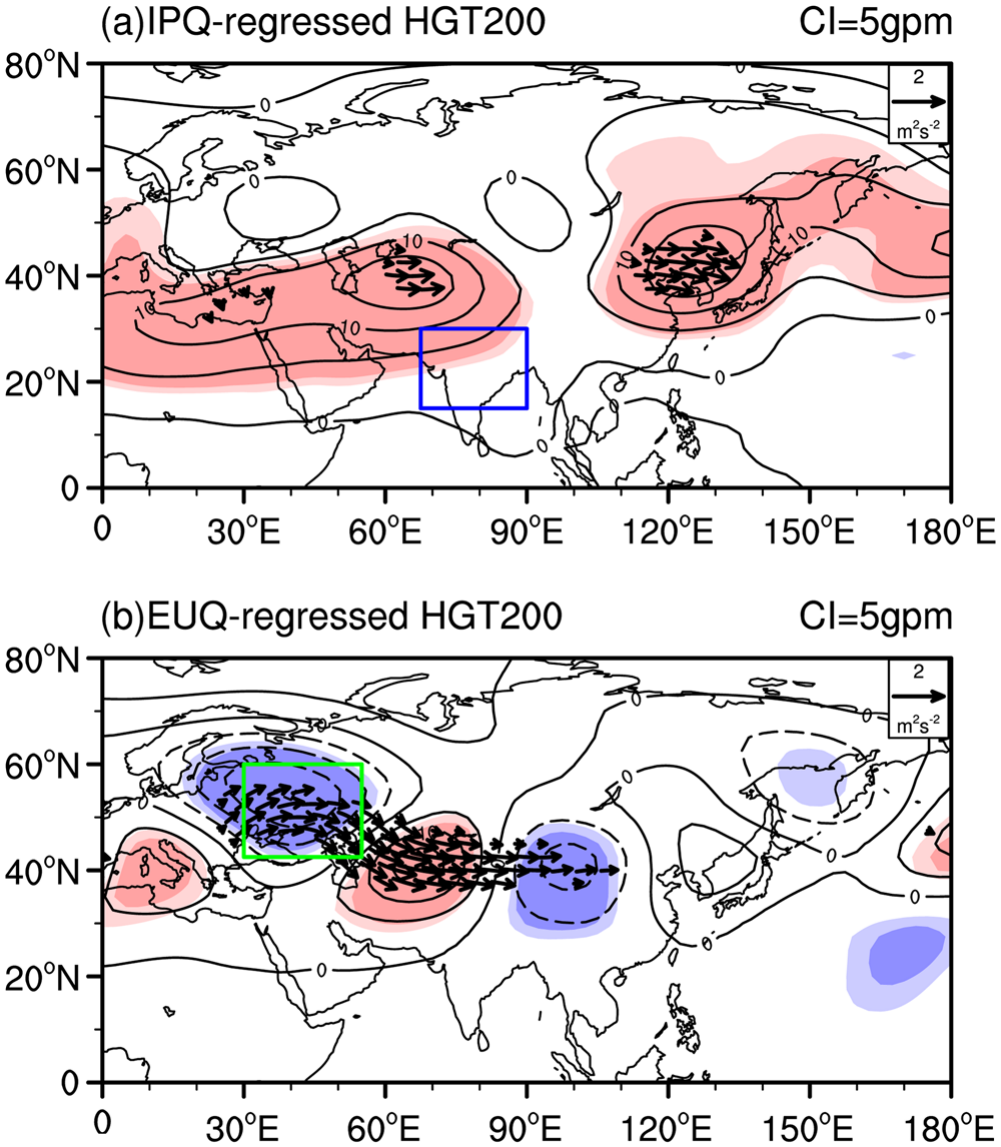
Regression of the 200 hPa geopotential height (black contour, where the CI is 5 gpm) and wave activity flux anomalies on the regional mean <*Q*_1_> index over the (**a**) Indian subcontinent (15°–30°N, 67.5°–90°E) and (**b**) East Europe (42.5°–60°N, 30°–55°E). The dark (light) shading in (**a,b**) indicates geopotential height anomalies significant at the 99% (95%) confidence level. The boxes in (**a,b**) are similar to those shown in Fig. 2. Wave activity fluxes are omitted when both directions are less than 0.5 m^2^ s^−2^ in (**a**,**b**). This Figure is created by the NCL^58^ v6.4.0 (http://www.ncl.ucar.edu/).

We further examined the 200 hPa geopotential height anomalies and wave activity flux in summer, as obtained by regression on the IPQ and EUQ (Fig. 3). The geopotential height related to the IPQ shows two centers of significant positive anomalies, with one over North Africa extending eastward toward the Iranian Plateau and the other over northeast Asia (Fig. 3a). Comparison of Figs. 1a and 3a suggests that significant positive (negative) geopotential height anomalies appear around the Iranian Plateau when above (below) normal <*Q*_1_> occurs over the northern Indian Peninsula, which would result in westward (eastward) shift of the center of the summer SAH. The 200 hPa geopotential height associated with the EUQ displays an atmospheric wave train similar to the SRP (Fig. 3b) and is also closely similar to that related to the SAHI (Fig. 1d). In particular, the pattern correlation between the 200 hPa geopotential height anomalies shown in Fig. 3b and Fig. 1d over 0°–80°N, 0°–180°E reaches 0.76. This confirms that the anomalous atmospheric heating around East Europe may play an important role in modulating the interannual variation in the SAH zonal location by inducing the Silk Road teleconnection-like atmospheric wave train. It is noted that the correlation coefficient between the IPQ and EUQ is about 0.23, which is statistically insignificant. In addition, Fig. S1a (please see supplementary) displays 200 hPa geopotential height anomalies regressed upon the EUQ_res index. Here, EUQ_res index is defined as the part of the EUQ that linearly unrelated to the IPQ. It is shown that spatial distributions of the 200 hPa geopotential height anomalies related to the EUQ and EUQ_res are highly similar (see Fig. S1a and Fig. 3b). Furthermore, Fig. S1b (please see supplementary) shows 200 hPa geopotential height anomalies regressed upon the IPQ_res index. IPQ_res index is defined as the part of the IPQ that linearly unrelated to the EUQ. Similarly, it is shown that spatial patterns of the 200 hPa geopotential height anomalies related to the IPQ remain similar after removing the impact of the EUQ (see Fig. S1b and Fig. 3a). Above evidences demonstrate that interannual variation of the EUQ is independent of the IPQ. The combined impacts of the EUQ and IPQ on the SAH is worth of further investigation.

**Figure 4:**
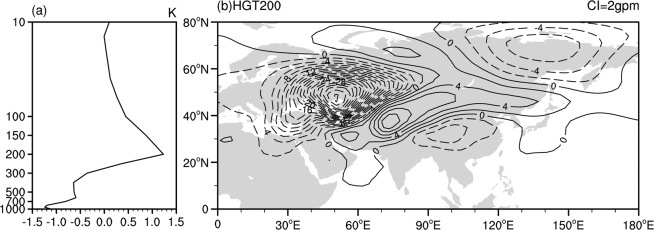
(**a**) Composite difference in the area-mean air temperature over East Europe (i.e., 42.5°–60°N, 30°–55°E) between the positive and negative phases of the EUQ; (**b**) 200 hPa height anomalies as a response to the prescribed diabatic heating over East Europe (i.e., 42.5°–60°N, 30°–55°E). This Figure is created by the NCL^58^ v6.4.0 (http://www.ncl.ucar.edu/).

The above observational evidence has indicated that the anomalous atmospheric heating around East Europe has a close relationship with the interannual variation in the SAH zonal location via triggering an atmospheric wave train. In this section, we further performed a numerical experiment to confirm the contributions of the atmospheric heating anomalies around East Europe to the zonal shift in the SAH by using the LBM. The basic state is prescribed as the summer mean for the period of 1948–2016 based on the NCEP-NCAR data. Figure 4a displays the composite difference in the air temperature averaged over East Europe between positive and negative years of the EUQ. Note that the positive and negative years of the EUQ are defined based on one standard deviation. Negative air temperature anomalies are observed below 300 hPa, with a maximum of approximately 925 hPa and a value of −1.24 K. In the following section, the vertical heating profile of the LBM experiment was set according to the distribution shown in Fig. 4a, which has Gamma distribution peaks at σ = 0.95 (approximately 900 hPa) over East Europe. In addition, heating has an idealized elliptical profile in the horizontal direction, which mimics the position and strength of heating over East Europe. The time integration is continued up to 30 days, and the results shown here are the mean from day 15 to 25. Figure 4b shows the 200 hPa height perturbation as a response to the prescribed diabatic heating anomalies over East Europe. As shown in Fig. 4b, significant negative geopotential height anomalies are seen over Europe and west China. In addition, pronounced positive geopotential height anomalies appear over the Iranian plateau. Spatial pattern of the geopotential height anomalies in Fig. 4b, to a large extent, is similar to that shown in Fig. 3b, although the negative geopotential height anomalies around Europe in the LBM cover larger areas and the negative geopotential height anomalies around west China shift slightly southward. It should be mentioned that previous studies^50,53^ also reported the strong coherence between positive (negative) geopotential height anomalies and positive (negative) surface temperature anomalies over Europe during positive (negative) phase of the SRP. It is clear that an atmospheric wave train induced propagation from East Europe to Southeast Asia, with a horizontal distribution similar to that related to the SAHI in Fig. 1d and associated with the EUQ in Fig. 3b. Therefore, the LBM experiment results verify that the anomalous atmospheric heating around East Europe can impact the interannual variation in the SAH zonal location by inducing an atmospheric wave train, with a structure similar to the SRP.

In addition, many previous studies^29,55,56^ have indicated that diabatic heating around the Indian Peninsula can impact the SRP and zonal shift of the SAH via observational analysis and numerical experiments. We further conduct numerical experiment to confirm role of diabatic heating around the northern Indian Peninsula in modulating the SPR and SAH via the LBM. The maximum heat forcing is 1.04 K/day over northern Indian Peninsula, and the vertical profile of the heating has a Gamma distribution which peaks at σ = 0.45 (about 400 hPa). The other settings are similar to the experiment related to the EUQ. Figure S2 (please see supplementary) presents the 200 hPa height perturbation as a response to the prescribed diabatic heating anomalies over northern Indian Peninsula. From the Fig. S2, large positive geopotential height anomalies with values above 1 gpm appear over northeast Africa extending eastward across Iranian Plateau to Okhotsk sea. Two centers of positive geopotential height anomalies are observed over the Iranian Plateau and the northern China, respectively, similar to that shown in Fig. 3a, although slight biases exist in the location of the centers. Above analysis further confirms that anomalous atmospheric heating over northern Indian Peninsula has a significant impact on the zonal shift of the SAH, consistent with previous studies^29,56^. Comparison of Fig. 4b and Fig. S2 indicates that EUQ impacts zonal shift of the SAH by inducing an atmospheric wave train similar to the Silk Road teleconnection pattern, while the IPQ impacts zonal shift of the SAH likely via a Rossby wave type atmospheric response.

## Conclusion and discussion

In this paper, we reveal that the <*Q*_1_> around East Europe has a close relation with the zonal shift in the SAH at the interannual scale. Specifically, when there are positive (negative) anomalies of <*Q*_1_> around East Europe, the SAH tends to shift westward (eastward). <*Q*_1_> around East Europe impacts the SAH zonal shift mainly through triggering an atmospheric wave train over the Eurasian subtropics by propagating eastward along the subtropical jet stream. In addition, it is found that the atmospheric wave train induced by the <*Q*_1_> around East Europe also resembles the Silk Road teleconnection pattern. The results of this study imply that anomalous atmospheric heating over East Europe is another important source besides the tropical SST anomalies for the interannual variation in the SAH zonal location. We examine possible connections of the diabatic heating around East Europe (i.e., represented by the EUQ) with several dominant ocean-atmosphere systems, including the ENSO (Nino3.4 index, defined as area-mean SST anomalies over 5°S-5°N and 120°−170°W), SST anomalies in the tropical Indian Ocean (TIO, defined as area-mean SST anomalies over 20°S-20°N and 40°–100°E), the NAO index, and Indian summer monsoon (ISM, defined as the difference of the 850 hPa zonal winds between regions of 5°–15°N, 40°–80°E and 20°–30°N, 70°–90°E following Wang *et al*.^57^). Correlation coefficients between the EUQ and the Nino3.4 index, TIO, NAO index, and ISM are - 0.11, 0.09, 0.19, and 0.07, respectively. All of the correlation coefficients are weak and statistically insignificant. This suggests that interannual variation of the diabatic heating around East Europe has a weak relationship with the tropical ENSO, Indian Ocean SST, NAO, and ISM. Interannual variation of the diabatic heating around East Europe may be related to the local atmospheric internal variability. More investigations are needed in the future to reveal the detailed factors for the interannual variation of the diabatic heating around East Europe.

We also calculated composite surface air temperature anomalies between the positive and negative phases of the EUQ (please see Supplementary Fig. S3a). Significant negative air temperature anomalies appear around East Europe, where significant positive <*Q*_1_> is located. Formation of the significant negative surface air temperature anomalies can be attributed to the horizontal temperature advection induced by anomalous winds. From the correlation pattern of the 850 hPa winds with the EUQ (please see Supplementary Fig. S3b), a significant cyclonic anomaly is seen over East Europe. The associated northerly wind anomalies to its west flank contribute to negative surface air temperature anomalies via carrying colder air from higher latitudes (see Fig. S3a,b). In addition, we further calculate the correlation pattern between the area-mean air temperature over East Europe and the 200 hPa geopotential height (please see Supplementary Fig. S3c). It shows that spatial structure of the 200 hPa geopotential height anomalies related to the area-mean air temperature over East Europe bears a close resemblance to that in the positive phase of SRP and negative phase of SAHI. The temporal correlation coefficient between the area-mean air temperature over East Europe and the SRP index (SAHI) is 0.6 (−0.38), both statistically significant. Hence, zonal variation of the SAH also has a close relationship with the area-mean surface air temperature in East Europe.

In addition, previous studies^20,23,31^ indicated that the tropical SST has an impact on the variation of the SAH. For example, Yang *et al*.^31^ reported that variation in the SAH zonal location is significantly impacted by SST anomalies in the TIO and Pacific. Huang *et al*.^20^ pointed out that the SAH tends to shift southward when SST in the TIO is warmer than normal. A question is whether impact of the atmospheric heating over East Europe on SAH is independent of the tropical SST-SAH relationship? To address this issue, we have calculated the correlation coefficients of the SAHI with the EUQ, Nino3.4 index, and TIO. Correlation coefficients of the SAHI with the EUQ, Nino3.4 index and TIO are 0.51, −0.38 and 0.057, respectively. This indicates that the interannual variation in the EUQ, Nino3.4 index and TIO can explain approximately 27%, 14%, and 0.3% of the interannual variation in the SAH zonal shift, respectively. Meanwhile, we remove the summer tropical Indian Ocean SST variance from the EUQ and SAH zonal shift index. Results show that, after removal of the TIO variance, the EUQ still has a significant correlation with the zonal shift index of SAH, with a correlation of about 0.55 (significant at the 99% confidence level). In addition, after removal of the TIO variance, spatial distribution of the 200hPa geopotential height anomalies related to the EUQ remains similar (please see Supplementary Fig. S4a and Fig. 4b). Furthermore, we have also calculated correlation of the EUQ with the SAH after removing the summer Nino3.4 index variance. Results indicate that the EUQ still has a pronounced relation with the SAH index after removal of the Nino3.4 index variance (r = 0.52, significant at the 99% confidence level). Similarly, spatial pattern the 200hPa geopotential height anomalies related to the EUQ also remain similar after removing impact of the El Niño (please see Supplementary Fig. S4b and Fig. 4b). This indicates that the impact of the atmospheric heating over East Europe is independent of the tropical Indian and Pacific SST. Nevertheless, due to the atmospheric heating over East Europe is less predictable, it may cause difficulty to predict the SAH zonal shift directly based on the atmospheric heating over East Europe. Prediction of the atmospheric heating over East Europe remains to be explored.

## Supplementary information

Supplementary information.
