# Supplementary material for: Potential impact of atmospheric heating over East Europe on the zonal shift in the South Asian high: the role of the Silk Road teleconnection
Source: Sci Rep. 2020 Apr 16;10:6543. doi: 10.1038/s41598-020-63364-2 (PMC7162852; doi:10.1038/s41598-020-63364-2)
Supplement: Supplementary file 1 — Supplementary information. [file 41598_2020_63364_MOESM1_ESM.docx]

*For submission to Scientific Reports*

Supplementary Information

**Potential impact of atmospheric heating over East Europe on the zonal shift in the South Asian high: the role of the Silk Road teleconnection**

Sixian Cen, Wen Chen, ShangfengChen,Yuyun Liu, Tianjiao Ma

**Supplementary Figure S1.** Regression maps of the 200 hPa geopotential height anomalies onto the (a) EUQ_res, and (b) IPQ_res. Definitions of the EUQ and IPQ are provided in the main text. EUQ_res index is defined as the part of the EUQ that linearly unrelated to the IPQ. IPQ_res index is defined as the part of the IPQ that linearly unrelated to the EUQ. The dark (light) shadings indicate geopotential height anomalies significant at the 99% (95%) confidence level. This Figure is created by the NCL^58^ v6.4.0 (<http://www.ncl.ucar.edu/>).

**Supplementary Figure S2.** 200 hPa height anomalies as a response to the prescribed diabatic heating over northern Indian Peninsula (i.e., 15°-30°N, 67.5°-90°E). This Figure is created by the NCL^58^ v6.4.0 (<http://www.ncl.ucar.edu/>).

**Supplementary Figure S3.** (a) Composite anomalies of the surface air temperature (unit: K) between the positive and negative phases of the region-averaged *<Q_1_>* over East Europe. (b) Correlations between the 850 hPa wind and the region-averaged *<Q_1_>* index over East Europe. (c) Correlations between the area-mean surface air temperature over East Europe and the 200 hPa geopotential height. Anomalies that are significantly different from zero at the 95% confidence level in (a) are marked with black dots. The vector is only drawn at the 95% confidence levels in (b). The dark (light) shadings in (c) indicate the correlation coefficient significant at the 99% (95%) confidence level. This Figure is created by the NCL^58^ v6.4.0 (<http://www.ncl.ucar.edu/>).

**Supplementary Figure S4.** Correlations of 200 hPa geopotential height (contours) with region-averaged anomalies of the *<Q_1_>* over East Europe (42.5^o^-60^o^N, 30^o^-55^o^E), but (a) removed the summer tropical Indian Ocean (TIO) variance, and (b) removed the summer EI Niño variance. The dark (light) shadings indicate the correlation coefficient significant at the 99% (95%) confidence level. This Figure is created by the NCL^58^ v6.4.0 (<http://www.ncl.ucar.edu/>).
